# Prospective randomized controlled trial comparing the efficacy and safety of Roux-en-Y gastric bypass and one-anastomosis gastric bypass (the RYSA trial): trial protocol and interim analysis

**DOI:** 10.1186/s13063-019-3898-y

**Published:** 2019-12-30

**Authors:** Tuure Saarinen, Sanna Meriläinen, Vesa Koivukangas, Kirsi Hannele Pietiläinen, Anne Juuti

**Affiliations:** 1 Department of Gastrointestinal Surgery, Helsinki University Hospital, Abdominal Center, Haartmaninkatu 4, 00029 HUS Helsinki, Finland; 2 Department of Surgery, Oulu University Hospital, Oulu, Finland; 3 Research Program for Clinical and Molecular Metabolism, Faculty of Medicine, University of Helsinki, Helsinki, Finland; 4 Department of Endocrinology, Helsinki University Hospital, Abdominal Center, Helsinki, Finland

**Keywords:** Obesity, Bariatric surgery, Metabolic surgery, Roux-en-Y gastric bypass, One-anastomosis gastric bypass

## Abstract

**Introduction:**

There is a lack of prospective studies comparing Roux-en-Y gastric bypass (RYGB) and one-anastomosis gastric bypass (OAGB). Also, the effects of bariatric surgery and weight loss need a deeper understanding through metabolic studies. We describe the trial protocol and interim analysis of a prospective randomized controlled study comparing RYGB and OAGB: the RYSA trial.

**Materials and methods:**

In total, 120 bariatric patients will be randomized between RYGB and OAGB in two academic centers. All patients will be followed up for 10 years with analysis and measurements of weight, comorbidities, blood tests, body composition and questionnaires. Extensive metabolic analyses (mixed meal tests, energy expenditure, biopsies of muscle and subcutaneous fat, urine, saliva and fecal samples) will be carried out in the Obesity Research Unit, University of Helsinki, for all patients treated at the Helsinki University Hospital (80 patients) at baseline, 6 months and 12 months. Bile reflux will be studied for the OAGB group at the Helsinki University Hospital at 6 months with gastroscopy and scintigraphy.

**Results:**

At an interim analysis at 3 months (half-way) through recruitment (30 RYGB and 30 OAGB patients) there have been no deaths and no intensive care unit admittances. One patient in both groups required additional gastroscopy, with anastomosis dilatation in the RYGB group but with no additional intervention in the OAGB group.

**Conclusion:**

The trial can be safely carried out. Recruitment is estimated to be complete by the end of 2019.

**Trial registration:**

Clinical Trials Identifier NCT02882685. Registered on August 30th 2016.

## Introduction

Surgery is the most effective treatment for morbid obesity, and gastric bypass has been considered a gold standard [1, 2]. Currently there are several viable options for performing a gastric bypass. Roux-en-Y gastric bypass (RYGB) has been modified from the bypass method published by Mason and Ito in the 1960s [3]. The hallmarks of RYGB are a small gastric pouch anastomosed to an alimentary limb and a separate biliopancreatic (BP) limb [4]. In 2001 Rutledge published a technique called mini-gastric bypass (MGB; later termed one-anastomosis gastric bypass (OAGB) or single-anastomosis gastric bypass (SAGB)) with a longer tubular-shaped pouch and a longer BP limb anastomosed directly into the distal end of the gastric pouch [5]. Since then, and according to the International Federation for the Surgery of Obesity and Metabolic Disorders 2018 statement on MGB/OAGB/SAGB procedures, a preferred term for all bypasses with a long gastric pouch and a long BP limb should be OAGB [6].

RYGB and OAGB have both shown excellent weight-loss results in many cohort series and prospective trials [7–9]. However, to our knowledge only two prospective randomized controlled trials comparing RYGB and OAGB have been published [10, 11]. It has been stated that OAGB would be at least as effective, faster to perform and less prone to complications such as internal herniations compared to RYGB [12]. On the other hand, it has been suggested that OAGB can cause bile reflux, a potential risk for premalignant Barrett’s metaplasia of the esophagus. OAGB has been proposed to have a more favorable effect on type 2 diabetes mellitus (T2DM) but might be more prone to nutritional deficiencies [6, 11]. The BP limb is usually longer in OAGB compared to RYGB, which most likely affects T2DM remission, but limb lengths have not been standardized in either procedure. Overall, there are a lot of unanswered questions when comparing RYGB and OAGB. A recent meta-analysis on this subject concluded that a larger sample size and multicenter randomized controlled trials are needed to compare the effectiveness and safety between these procedures [13].

Bariatric surgery induces changes in the entire body including body composition, bile acid metabolism, energy expenditure, cellular metabolism, gut microbiota, chronic stress, vitamin and electrolyte homeostasis, glucose tolerance and lipid metabolism, as well as eating behavior, physical activity, self-image, social relationships and quality of life. The mechanisms of these changes are not fully understood, and neither are the differences in the underlying mechanisms of RYGB and OAGB. A comprehensive approach to obesity management is needed in order to be able to tailor a treatment for each patient.

Our objective is to compare outcomes of RYGB and OAGB procedures and to study comprehensively all changes in obesity-related conditions in a prospective study where we randomize between RYGB and OAGB.

Here we describe the trial protocol for a randomized controlled trial, the Roux-en-Y Gastric Bypass vs Single-Anastomosis Gastric Bypass (RYSA) trial, with an interim analysis regarding safety and trial progression.

## Materials and methods

### Aims of the study

The primary outcome is weight loss 2 years after the operation. Weight loss is measured as percentage excess weight loss (%EWL) and percentage total weight loss (%TWL). %EWL is calculated as a percentage of lost excess weight since the preoperative visit compared to an ideal weight with a body mass index (BMI) of 25 kg/m^2^. Weight loss will also be analyzed as a grouping variable according to different categories of weight loss to compare responders and nonresponders to bariatric surgery. For EWL% we use the categories 0–24.99%, 25–49.99%, 50–74.99% and ≥75%, and for TWL% we use the categories <10%, 10–19.99%, 20–29.99%, 30–39.99%, ≥40% to determine participants with different amounts of weight loss achieved.

The secondary outcomes to be measured at the time points of 6 months, 12 months, 24 months, 5 years and 10 years are as follows:

- Weight loss (%EWL, %TWL)
- Complications (any complication requiring any intervention or a prolonged hospital stay or additional outpatient visits)
- Perioperative factors (operation time, hospital stay, nausea, ability to take fluids and mobilization)
- Remission of comorbidities (including, for example, T2DM, hypertension, nonalcoholic fatty liver disease); remission is defined as normalization of measured values without medication
- Changes in nutritional, metabolic and safety laboratory parameters (e.g., hemoglobin, vitamins, albumin, electrolytes, liver enzymes, lipids)
- Glucose tolerance and insulin response during an oral glucose tolerance test (OGTT) or meal test
- Continuous glucose monitoring (CGM)
- Changes in body composition (whole body fat with dual-energy x-ray absorptiometry (DEXA) and bioelectrical impedance (BIA), lean mass (DEXA and BIA), bone mineral mass and density (DEXA), subcutaneous fat (magnetic resonance imaging (MRI)), intra-abdominal fat (MRI), liver fat (magnetic resonance spectroscopy (MRS))
- Bile reflux at 6 months (for the OAGB group at Helsinki University Hospital (HUH))
- Tendency for urolithiasis
- Changes in cortisol and other hormone metabolism
- Changes in blood and tissue (adipose tissue, muscle) transcriptomics, proteomics, metabolomics
- Changes in tissue (adipose tissue, muscle) mitochondrial activities
- Changes in gut and saliva microbiota and bile acid metabolism
- Changes in quality of life and lifestyle and gastrointestinal symptoms

Drop-out and lost-to-follow-up patients will be included in the analyses with measurements and values that have been obtained prior to drop-out. A 10% drop-out rate has been included in the power calculation regarding the primary outcome. Patients are regarded as part of their original treatment group according to randomization on an intention-to-treat basis.

An interim analysis regarding safety and trial progression has been performed after half of the patients have been randomized and undergone surgery.

### Sample size

This study is designed as a superiority trial based on previous cohort series. According to power calculations finding a difference of 10 in %EWL with the assumptions of mean %EWL = 60 (standard deviation (SD) = 17) in the RYGB group, with alpha = 0.05, 50 patients in each group would be sufficient to reach the power of 80%. If a drop-out of 10% is taken into account, this would mean 55 patients per group. Power calculations were performed by simulation using the Mann-Whitney-Wilcoxon test (PASS v13.0, NCSS Inc., Kaysville, Utah, USA). Since there was a lack of published data on comparisons between these two operations at the time of designing the study, we decided to recruit 60 patients for both treatment groups in order to have sufficient statistical power regarding the primary end point (10% difference in %EWL at 2 years between the groups).

### Inclusion criteria

Inclusion criteria were:

- Age >18 years
- BMI ≥35 kg/m^2^
- Eligible for gastric bypass surgery according to national treatment guidelines
- Willingness to participate in this trial

### Exclusion criteria

Exclusion criteria were:

- Anemia (hemoglobin <120 g/l)
- Pregnancy or lactation
- For MRI/MRS imaging: metal objects in the body or claustrophobia
- Endoscopic evidence of hiatal hernia, reflux esophagitis or Barret’s esophagus
- Any other condition that, in the opinion of the investigator, could create a hazard for the safety of the participant, endanger the study procedures or interfere with the interpretation of study results
- Lack of consent

### Study sites and randomization

The trial is being carried out in two academic centers: HUH in collaboration with the University of Helsinki, and Oulu University Hospital (OUH). Both bariatric centers (HUH and OUH) are university hospitals that are tertiary referral bariatric centers with a catchment area of around one million people in Finland. Metabolic studies for patients in HUH are conducted by the Research Program for Clinical and Molecular Metabolism, University of Helsinki.

**Fig. 1:**
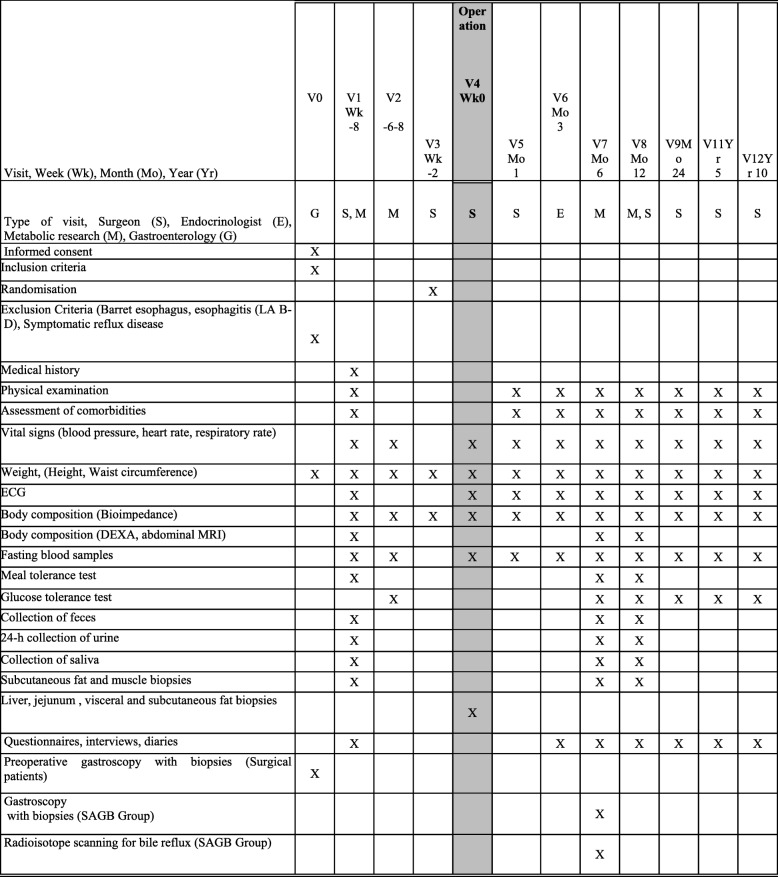
Trial schedule. DEXA dual-energy x-ray absorptiometry, ECG electrocardiogram, LA Los Angeles (classification), MRI magnetic resonance imaging, SAGB single-anastomosis gastric bypass, V visit

The trial is carried out according to the Consolidated Standards of Reporting Trials (CONSORT) statement. The trial schedule is shown in Fig. 1. Surgeons performing the intervention recruit eligible patients at a preoperative visit (study baseline) and randomize them according to protocol. This is an open-label study and the patients and surgeons are informed of the randomization outcome. Outcome analyses are also performed without blinding. Randomization is carried out as follows: 120 sheets of paper stating either “Single Anastomosis Gastric Bypass” or “Roux-en-Y Gastric Bypass” are divided into groups of four and six containing equal numbers of both outcomes and then sealed into opaque envelopes. These groups of opaque envelopes are distributed to both centers (80 envelopes for HUH and 40 envelopes for OUH) and then divided into two batches (for patients with diabetes and those without). The goal of this allocation sequence is to have equal numbers of patients in both groups at both centers and also an equal number of patients with diabetes in both groups at both centers.

### Interventions

When our current randomized study was designed there was no consensus regarding the name for the OAGB procedure in the bariatric surgery community. Hence, we then decided to use the name SAGB and we named the trial RYSA (Roux-en-Y Gastric Bypass vs Single-Anastomosis Gastric Bypass).

#### Roux-en-Y gastric bypass

**Fig. 2:**
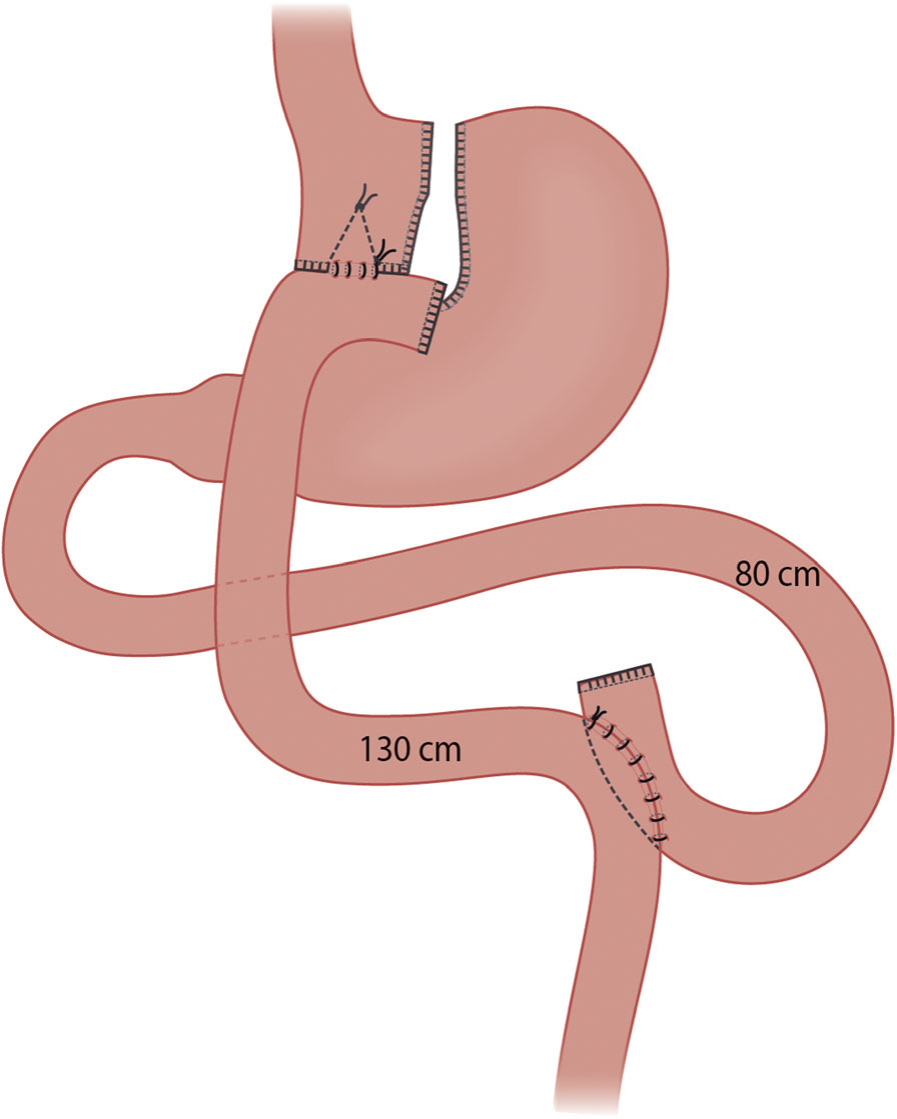
Illustration of the Roux-en-Y gastric bypass technique

After insufflation with CO_2_ reaching 15 mmHg intra-abdominal pressure, a standard four-port laparoscopy with a subxiphoidal liver retractor in place is carried out. Biopsies of subcutaneous fat, the omentum and liver are taken (at HUH). At 5 cm below the gastroesophageal junction, an approximate 20- to 40-ml small pouch is created with linear staplers. The omentum is divided and an antecolic 80-cm BP limb is measured with a marked dissector and anastomosed end-to-side with a linear 45-mm stapler and the anterior defect is sutured in two layers with a braided absorbable 2–0 running suture. A 130-cm alimentary limb is measured with a marked dissector and enteroanastomosis is created with a 60-mm or 45-mm (at the HUH and OUH, respectively) linear stapler and the remaining anterior defect is sutured in one layer with a braded absorbable 2–0 running suture. The ends of both anastomotic staple lines are secured with an extra stich. The connection between the anastomoses is divided with two linear staplers and a biopsy of the small bowel is obtained between the staple lines (at HUH). The mesenteric defect and Petersen’s space are closed with titanium clips or a slowly (180 days) absorbable barbed running suture (at the HUH and OUH, respectively) (Fig. 2).

#### One-anastomosis gastric bypass

**Fig. 3:**
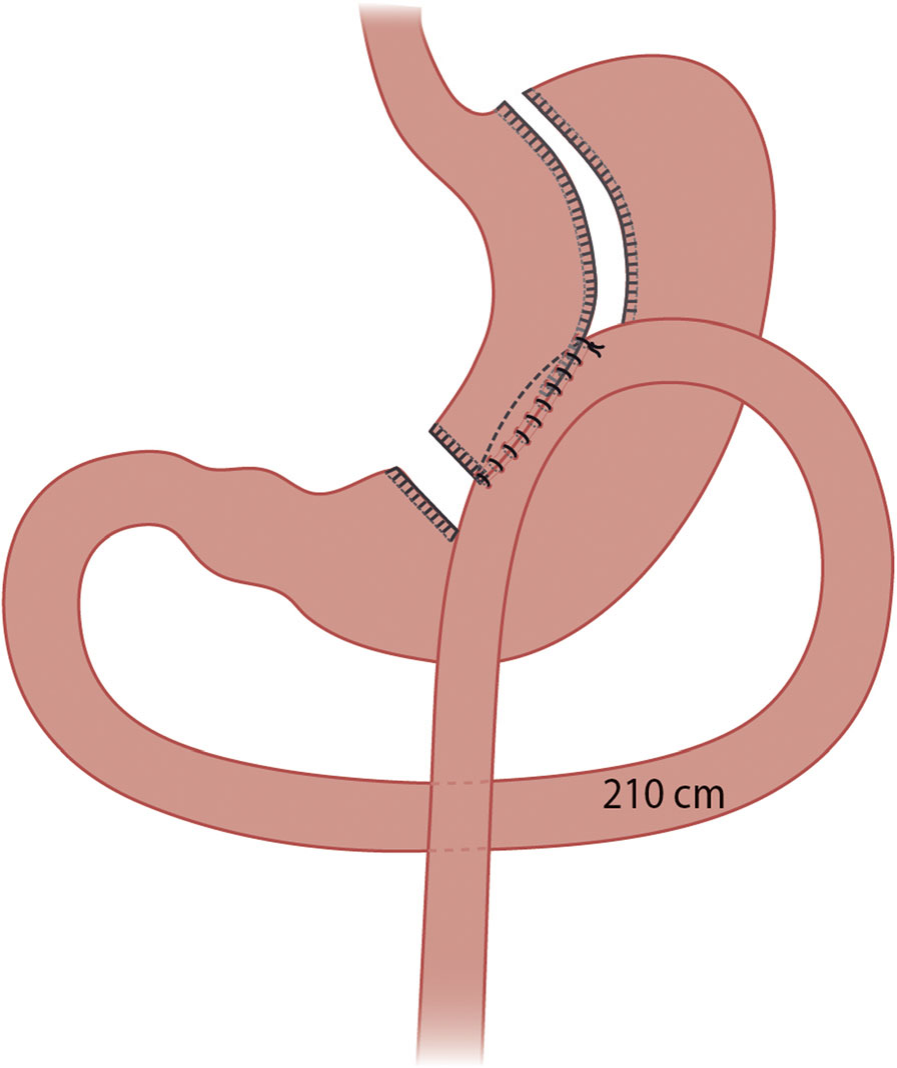
Illustration of the one-anastomosis gastric bypass technique

After insufflation with CO_2_ reaching 15 mmHg intra-abdominal pressure, a standard four-port laparoscopy with a subxiphoidal liver retractor in place is carried out. Biopsies of subcutaneous fat, the omentum and liver are taken (at HUH). A long gastric pouch is created with linear staplers starting horizontally at the crow’s foot and continuing towards the angle of this along a 38-Fr bougie. An antecolic 210-cm BP limb is measured with a marked dissector and anastomosed with a linear 45-mm stapler side-to-side with the distal end of the pouch. The remaining defect is sutured in two layers with a braided absorbable 2–0 running suture beginning and ending 2 cm proximal to the stapled anastomosis, thus securing the BP limb’s position parallel to the pouch (Fig. 3).

We chose to do a 210-cm BP limb in OAGB and 80-cm (BP) and 130-cm (alimentary) limbs in RYGB to obtain equally long bypassed intestine in both groups.

### Inpatient follow-up

After the operation all patients are monitored for 2 h in the operation unit and then transferred to the ward, where blood pressure, pulse, heart rate, temperature, and blood glucose are measured and documented together with pain, nausea and fluid intake. Patients are mobilized and fluids are given as soon as possible. Patients are discharged on the first or second postoperative day if there are no abnormal symptoms or suspicion of complications (elevated heart rate, fever, pain, nausea, vomiting, low hemoglobin). Fluid intake must be at least 1000 ml/day and the patient must be sufficiently mobilized and feel fit to go home. All patients are prescribed multivitamins (Multivita Plus®) once a day, calcium carbonate and vitamin D 1000 mg + 20 IU/day, and vitamin B12 1 mg/day. Women of fertile age are also prescribed iron substitution 100 mg/day. Other prescriptions include a proton-pump inhibitor (pantoprazole 40 mg/day) for 3 months (at HUH) and the antithrombotic agent enoxaparin 40 mg/day subcutaneously for 10 days. Paracetamol 1000 mg three times a day, metamizole + pitofenone 500/5 mg three times a day and tramadol 50 mg three times a day are prescribed for pain.

### Preoperative work-up and outpatient follow-up

#### Helsinki University Hospital and Oulu University Hospital

At 8 weeks prior to the operation (the baseline of the study), all patients are evaluated for eligibility for surgical treatment according to the Finnish guidelines for management of obesity and local protocols by an endocrinologist, surgeon, dietitian and anesthesiologist. This includes a thorough medical check-up with laboratory tests, BIA, assessment of obstructive sleep apnea and a gastroscopy. If the inclusion criteria are met and no exclusion criteria are found, patients can be recruited to this study. Written consent is received for all study patients. Together with extensive blood tests, we perform an OGTT to identify patients with impaired glucose tolerance or T2DM. Preoperatively, all patients also fill in questionnaires to assess diseases, medications, lifestyle and quality of life.

At 1 month after the operation, postoperative recovery and comorbidities are assessed together with blood tests and BIA.

At 3 months all patients meet with an internist or endocrinologist, and nutritional, metabolic and safety laboratory tests are evaluated and medications for comorbidities are reviewed and optimized (at HUH). At OUH, laboratory tests are analyzed, and patients are contacted via telephone.

At 6 months a gastroscopy with comprehensive biopsies of the gastric tube, gastroesophageal junction and esophagus as well as a radioisotope scanning for the detection of bile reflux are performed (for the OAGB group at HUH).

At 6 months,12 months, 24 months, 5 years and 10 years BIA measurement and OGTT are repeated according to the same protocol as prior to the operation. Accordingly, nutritional, metabolic and safety laboratory tests are again performed. All patients also fill in questionnaires and food diaries (at HUH). At OUH, all patients meet an endocrinologist, and nutritional, metabolic and safety laboratory tests are evaluated and medications for comorbidities are reviewed and optimized.

#### University of Helsinki Obesity Research Unit

At 8 weeks before the operation, metabolic examinations and a meal tolerance test are performed for all patients at HUH. CGM is performed using the Abbott Freestyle Libre device. Biopsies of subcutaneous fat, skin and a muscle tissue (vastus lateralis muscle) are taken under local anesthesia. Blood and diurnal urinary samples are collected in order to analyze indicators of nutritional status, metabolic and safety parameters, glucose tolerance, cortisol and other hormone metabolism, bile acid metabolism, calcium homeostasis and a tendency for urolithiasis. Salivary bilirubin and microbiota and gut microbiota are analyzed from saliva and feces samples, respectively. Omics analyses are performed from blood and tissue specimens and mitochondria-specific measurements taken from tissue biopsies. DEXA and abdominal MRI and MRS are used for analysis of the distribution of fat tissue. The participants also fill out questionnaires and diaries, and they are interviewed on health, symptoms, lifestyle, and quality of life.

The exact same protocol is repeated for all patients at HUH at 6 and 12 months after the operation.

### Body composition studies

Each participant’s weight, height, and waist and hip circumference are measured and BMI and waist to hip ratio are calculated. Body composition is analyzed by BIA, DEXA, MRI, and proton MRS.

#### Bioelectrical impedance

The body water, body fat and amount of lean tissue are calculated by measuring electrical impedance (Tanita MC-980).

#### DEXA

The bone mineral content, fat mass, and fat-free mass are determined using a Lunar Prodigy whole-body scanner (GE Medical Systems, Madison, WI).

#### MRI and MRS

Body fat distribution and liver fat content are analyzed using MRI and MRS. The imaging and data analysis procedures have been described elsewhere [14].

### Indirect calorimetry

Indirect calorimetry (Cosmed Q-NRG) is used to estimate the basal metabolic rate from measurements of O_2_ consumption and CO_2_ production. This is measured with the patient lying supine in bed and breathing calmly and regularly in the canopy with a constant air flow (to be adjusted to give O_2_ and CO_2_ concentrations within the workable range).

### Fasting blood samples

Routine laboratory tests, including a complete blood count, and levels of vitamins, potassium, sodium, copper, selenium, zinc, magnesium, calcium, lactate, creatine kinase, pyruvate, creatinine, glycated hemoglobin A1c, cholesterol, low-density lipoprotein cholesterol, high-density lipoprotein cholesterol and triglycerides, plus a measure of thyroid function (with thyroid-stimulating hormone), liver enzymes (aspartate aminotransferase, alanine transferase, γ-glutamyltransferase, alkaline phosphatase, bilirubin), and synthesis markers of the liver (thromboplastin time, albumin) are taken. Lipoprotein fractions are separated for measurement of chylomicrons, high-density lipoprotein and low-density lipoprotein subspecies. In addition, metabolic markers such as cytokines (e.g., high-sensitivity C-reactive protein), systemic global metabolites (metabolomics, lipidomics, proteomics) and bile acids are analyzed. Investigation of hemostasis activity, coagulation parameters, platelet activity and function, as well as thrombin formation capacity in platelet-free and platelet-rich plasma are performed. Markers of chronic stress (e.g., copeptin, cortisol, cortisol metabolites, aldosterone, renin, metanephrine, normetanephrine) and calcium homeostasis (e.g., urate, phosphorus, intact parathyroid hormone) are also measured.

### MMT and OGTT

A mixed meal test (MMT) and OGTT are performed after an overnight (10-h) fast. During the MMT the patients eat a caloric-rich, partially liquid meal of 2620 kJ with a balanced distribution of fat (24 g), carbohydrates (76 g) and protein (24 g) (Resource® 2.5 Compact, Nestle Health Science). A fasting blood sample is collected before ingestion of the meal, and postmeal samples are collected at 15, 30, 60, 120, 180, 240 and 360 min for measurement of glucose, insulin, c-peptide, incretins, lipids and lipoproteins, appetite-regulating hormones and metabolites.

Additionally, a standard OGTT is performed with similar measurements as in the MMT. A fasting blood sample is collected after which patients take a 75-g oral glucose dose. Postglucose time points for sample collection are 0, 30, 60, 120, and 180 min.

### Saliva, urine and fecal samples

Saliva is collected after stimulation by chewing parafilm. Saliva samples are used for microbiota and metabolomics analyses.

Urine is collected for 24 h in free-living conditions at home. Urine samples are used to measure urinary albumin, urea, creatinine, sodium, potassium and magnesium, and for metabolomics analyses. Markers of chronic stress (e.g., cortisol, cortisol metabolites, aldosterone, adrenalin metabolites) and calcium homeostasis (e.g., calcium, citrate, urate, oxalate, phosphorus) are measured. Furthermore, an additional urine sample is collected on a study morning to perform a pregnancy test for females of childbearing age.

Feces are collected either at the study center or at home and stored at –80 °C within 24 h. Feces samples are used for metagenome sequencing and fecal metabolites, including fatty acid composition.

### Fat, muscle and skin biopsy

A subcutaneous fat biopsy by liposuction (approximately 3 g) is taken under local anesthesia from the abdominal area. One part of the sample is immediately frozen and stored in liquid nitrogen or −80 °C until used for transcriptional, protein or other biochemical analyses, and another part is further prepared for isolation of adipocytes and stroma vascular fraction (SVF) cells. Fat cell size is determined from fresh adipocytes. Part of the SVF is prepared for cell culture. Fat is also stored in paraffin for future immunohistochemical analysis.

A needle muscle biopsy (~50 mg) is taken under local anesthesia and sterile conditions from the vastus lateralis muscle with a Bergström needle. Part of the sample is immediately frozen in liquid nitrogen after excision and stored at –80 °C until used for transcriptional, protein or other biochemical analyses. Other parts are prepared for histological staining and electron microscopy and for myoblast culture.

A skin biopsy (approximately 1 × 1 cm) is taken from the abdomen before the fat biopsy. The biopsy specimen is processed for a fibroblast culture.

Using the adipose tissue and muscle biopsies, and also from the isolated SVF and myoblast cells, we perform a comprehensive set of analyses measuring mitochondrial biogenesis and function.

### Induced pluripotent stem cells, leukocytes and other blood cells

Leucocytes, preadipocytes, myoblasts and fibroblasts can be used for the production of induced pluripotent stem cells. Leukocytes and red blood cells are also extracted and stored live for future analyses, and extraction of DNA and RNA.

### Genetic, epigenetic, transcriptomic analyses and other omics analyses

DNA is isolated from a whole blood sample, tissue samples, feces and saliva. Genetic studies including genome-wide scans and sequencing technologies are performed to combine information obtained from genome-wide transcriptomics analyses of target tissues (adipose, skin, muscle, liver, gut, and so forth). Additionally, we will measure the mitochondrial DNA copy number of the tissues as an estimate of the mitochondrial amount. RNA isolated from blood, adipose tissue, muscle, skin or their cultures is used to study global tissue-specific RNA profiles. Epigenetic profiling of the tissues may include whole genome-scale methylation techniques.

Blood samples, tissue biopsies and urine samples are used for other omics analyses such as metabolomics, lipidomics and proteomics analyses. Saliva and fecal samples are used for microbiota analyses.

### Continuous glucose monitoring

CGM is carried out with the Freestyle Libre system (Abbott). The sensor is applied on the back of the upper arm and worn for 14 days per time point, during which all patients keep a food diary for 3 days.

### Hepatobiliary scintigraphy

Bile reflux is investigated with hepatobiliary scintigraphy as described elsewhere [15]. Scintigraphy is performed for all patient in the OAGB group of the HUH (40 patients) 6 months after the operation.

### Endoscopic and histological assessment

All patients undergo a gastroscopy with biopsies from the duodenum, antrum, corpus and gastroesophageal junction as a part of preoperative assessment. During the follow-up all OAGB patients at HUH are invited for a gastroscopy 6 months after OAGB to assess endoscopic signs of reflux. Gastroscopies are performed without sedation with a flexible endoscope (Olympus Q190, Tokyo, Japan). Mucosal biopsies are obtained from the jejunum, anastomosis, gastric pouch in 2-cm intervals, cardia and esophagus. Biopsy specimen are prepared with serial sections (5 μm thick) from formalin-fixed and paraffin-embedded biopsy specimen after hematoxylin and eosin and Alcian blue/periodic acid–Schiff staining.

### Questionnaires and food diaries

All patients fill out questionnaires that extensively survey their quality of life, physical activity, social activity, gastrointestinal symptoms, eating behavior, sleep, mental state and general health status. A food diary is additionally kept for 3 days to measure nutritional intake for patients operated on at HUH.

### Ethical approval and informed consent

All procedures performed in studies involving human participants are in accordance with the ethical standards of the Institutional Research Committee and with the 1964 Helsinki Declaration and its later amendments or comparable ethical standards. The trial has been reviewed by Helsinki University Hospital ethics committee (HUS/1706/2016) and approved by the Helsinki University Hospital Research Review Board (HUS269/2017). The trial is registered at www.clinicaltrials.gov (NCT02882685. Informed consent was obtained from all individual participants.

### Statistical analyses

Normally distributed variables will be expressed as the mean and SD, and non-normally distributed variables will be expressed as the median and interquartile range; categorical variables will be expressed as the number and percentage.

To test the group differences (OAGB vs RYGB) in the primary and secondary endpoints, we will use the Student’s *t* test for continuous normally distributed variables, the Mann–Whitney *U* test for continuous non-normally distributed data, and the chi-squared test or Fisher’s exact test for categorical variables. Additionally, we will conduct multivariable analysis with generalized mixed linear regression or logistic regression models with adjustment for possible confounding factors. The statistical analyses will be conducted on an intention-to-treat basis. A *P* value < 0.05 will be considered statistically significant.

## Interim results

According to our study design, we have made an interim analysis half-way through recruitment to check that there are no serious issues regarding safety according to the Clavien–Dindo classification [16] or problems regarding sample handling or data collection.

Between November 2016 and May 2018, 60 patients (30 RYGB and 30 OAGB) were randomized and operated on according to our study protocol.

Baseline BMI (given as median ± SD) was 44.0 ± 5.9 kg/m^2^ in the RYGB group and 44.9 ± 5.5 kg/m^2^ in the OAGB group. There were 20 women in the RYGB group and 21 in the OAGB group. The number of patients with T2DM at baseline was 13 in the RYGB group and 11 in the OAGB group and the median ± SD T2DM duration was 3.0 ± 5.9 years and 7.0 ± 4.9 years in the RYGB and OAGB groups, respectively. Other baseline characteristics are given in Table 1.

**Table 1:** Baseline characteristics

	Female (% of participants)	T2DM (% of participants)	T2DM duration, years (median ± SD)	HT (% of participants)	Dyslipidemia (% of participants)	OSA (% of participants)	Arthrosis (% of participants)
RYGB (*n* = 30)	20	13	3.0 ± 5.9)	18	8	12	16
OAGB (*n* = 30)	21	11	7.0 ± 4.9)	16	8	12	15

*HT* hypertension, *OAGB* one-anastomosis gastric bypass, *OSA* obstructive sleep apnea, *RYGB* Roux-en-Y gastric bypass, *T2DM* type 2 diabetes mellitus

During the first 3 months of follow-up there have been no deaths or reoperations in either group or the need for admittance to the intensive care unit. In both groups one patient has undergone additional gastroscopy due to eating difficulties (Clavien–Dindo class IIIa). The patient in the OAGB group had a normal esophagogastroscopy finding but the patient in the RYGB group needed endoscopic dilatation of the gastroenteral anastomosis. One patient in the RYGB group stayed one extra night on the ward after the operation due to low hemoglobin, but no transfusion or intervention was required (Clavien–Dindo Class I). One patient in the RYGB group had an incisional seroma (Clavien–Dindo class I). All other patients were discharged on the first or second postoperative day.

There have not been any significant problems with sample collection or data handling.

## Conclusion

Obesity is a multifactorial disease and treatment needs to be aimed at all aspects of the metabolic syndrome. Ever since the early days of bariatric surgery there has been enormous interest in finding out which is the optimal procedure. Each operation has its pros and cons and the definition of what is optimal is not clear. First, we need to discover the true underlying mechanisms of surgery-induced weight loss and remission of comorbidities and which of these mechanisms are related to a specific surgery and not just weight loss per se. Many studies have shown the metabolic effects of bariatric surgery and nowadays it is more appropriate to talk about metabolic surgery. The comparison of RYGB with OAGB has for a long time been a matter of opinion since there have not been enough data from randomized controlled trials, and both techniques have shown great results [9].

Our current study takes on a comprehensive approach to the entire concept of metabolic surgery and weight loss.

First, we are comparing RYGB and OAGB in a randomized controlled setting to find out whether there is a difference in weight loss, postoperative symptoms, adverse effects, comorbidity remission and quality of life between the procedures. We intend that any small differences between these techniques will help us understand which patient would benefit from a certain technique. Our study protocol includes a thorough follow-up with regular outpatient visits including blood analyses, body composition measurements and questionnaires. Bile reflux is measured with a specific scintigraphic method which we have previously tested in a pilot series [15]. In the current study, the scan is prolonged to 90 min to view the entire potential bile exposure of the gastric tube and esophagus. The risk of urolithiasis is analyzed from diurnal urine samples, blood analyses and DEXA measurements.

Second, we want to discover the metabolic effects of surgery and weight loss at a cellular level by measuring changes in mitochondrial activity and the interplay between gut hormones, bile acids, gut microbiota and the regulation of glucose homeostasis. We use novel analytic mechanisms for measuring energy expenditure and mitochondrial activity. Gut hormone and bile acid responses to meal stimulation are measured and glucose homeostasis is studied with CGM and repeated OGTT during the follow-up.

There might not be only one optimal metabolic surgery technique and, once we understand what obesity really is about at a cellular level and what changes are needed and how they are met in order to reach goals of obesity management, we can tailor our treatments to each patient.

The trial is limited due to the small number of patients and hence may be underpowered regarding some of the secondary outcomes. A second limitation is that both centers are more experienced in the RYGB technique, which is likely to have an effect when comparing operation duration. However, this should not interfere with other outcomes.

### Trial status

This trial protocol is version 6.3, 12 July 2015. According to the interim analysis the trial can be completed safely. Recruitment started on 13 September 2016 and is estimated to be completed in November 2019.

## Supplementary information

**Additional file 1.** SPIRIT 2013 checklist

## Data Availability

Not applicable.

## Abbreviations

%EWL: Percentage excess weight loss
%TWL: Percentage total weight loss
BIA: Bioelectrical impedance
BMI: Body mass index
BP: biliopancreatic
CGM: Continuous glucose monitoring
DEXA: Dual-energy x-ray absorptiometry
HUH: Helsinki University Hospital
MGB: Mini-gastric bypass
MMT: Mixed meal test
MRI: Magnetic resonance imaging
MRS: Magnetic resonance spectroscopy
OAGB: One-anastomosis gastric bypass
OGTT: Oral glucose tolerance test
OUH: Oulu University Hospital
RYGB: Roux-en-Y gastric bypass
SAGB: Single-anastomosis gastric bypass
SD: Standard deviation
SVF: Stroma vascular fraction
T2DM: Type 2 diabetes mellitus
